# Digitalization and digital transformation in higher education: A bibliometric analysis

**DOI:** 10.3389/fpsyg.2022.1081595

**Published:** 2022-12-02

**Authors:** Vicente Díaz-García, Antonio Montero-Navarro, José-Luis Rodríguez-Sánchez, Rocío Gallego-Losada

**Affiliations:** ^1^Facultad de Ciencias Jurídicas y Sociales, Universidad Rey Juan Carlos, Paseo de los Artilleros, Madrid, Spain; ^2^ESIC Business & Marketing School, Pozuelo de Alarcón, Madrid, Spain; ^3^Department of Business Economics (Adm., Dir. and Org.), Applied Economics II and Fundamentals of Economic Analysis, Facultad de Ciencias Jurídicas y Sociales, Universidad Rey Juan Carlos, Paseo de los Artilleros, Madrid, Spain; ^4^Department of Financial Economy and Accountancy, Facultad de Ciencias Jurídicas y Sociales, Universidad Rey Juan Carlos, Paseo de los Artilleros, Madrid, Spain

**Keywords:** digitalization, digital transformation, cultural change, bibliometric analysis, resistance to change, research trends, higher education institutions

## Abstract

The new paradigms that are emerging because of technological and social advances derived from the massive use of information and communication technologies (ICTs) are generating a transformative process that is modifying all economic sectors, and education is no exception. Higher Education Institutions (HEIs) are carrying out such transformation, reacting to the need of adaptation to this new reality, experiencing a complete cultural change that is challenging the attitudes, actions and values shared by the members and stakeholders of these organizations. In order to analyze the scientific literature about this topic, a bibliometric analysis has been carried out covering the period 1900–2021, considering a sample of 469 articles included in the Web of Science (WoS) database. The results show the multidisciplinary nature of the topic, including articles published in different areas, as well as its close link with aspects such as innovation, governance and agile methodologies. Finally, this study highlights the main lines of research that could attract more attention in the immediate future.

## Introduction

In the last years, the unstoppable development of information and communication technologies (ICTs) has given birth to what has been called the digital age or Industry 4.0. These technological advances are dramatically changing most fields of our day to day lives, as well as the dynamics of social and economic relations. The academic literature has often called this phenomenon *Fourth Industrial Revolution* (Xu et al., 2018), a change boosted by the critical role of digitalization and new technologies. Digital development has implications for the Sustainable Development Goals (SDGs) set out in the United Nations 2030 Agenda: countries, institutions and organizations should commit with the goal of reducing the digital divide to avoid the potential negative effects of digital exclusion (Kulkarni and Ghosh, 2021).

Companies often define technological innovation strategies based on the development and use of ICTs, focusing on infrastructure management (Matt et al., 2015). This causes a limited impact when it comes to achieving the creation of new scenarios and value propositions. So, just using ICTs, even squeezing their maximum performance, may provide organizations with scarce outcomes.

So, digitally transforming an organization is much more than just digitalizing it. It is the result of an organizational change where people, processes and the entire business model understand technology as a tool to generate value among its consumers and collaborators (Schwab, 2016). According to Demirkan et al. (2016: 14), DT is “the profound and accelerating transformation of business activities, processes, competencies, and models to fully leverage the changes and opportunities brought by digital technologies and their impact across society in a strategic and prioritized way.” Westerman et al. (2014) define the DT of an organization as the use of digital technologies to radically improve its performance and scope.

We are facing a disruptive change that will affect all organizations and their professionals (Schwab, 2016). It can generate resistance due to emotional, cognitive and behavioral reasons. To reduce this resistance, it is necessary to define an appropriate strategy to address it, with leaders being responsible for clearly communicating the need for change and encouraging professionals to participate in the project. By reducing resistance, an organization’s performance throughout the process will increase (Perides et al., 2020). Organizations are thus facing massive changes in work design and leadership, pushing new attitudes, values and ideas, which results in a complete cultural change. The new job profiles required are those that are adapted to the new positions. These workers will demand a new type of leadership oriented towards the relationships between people: more teamwork, more networked structures and greater need for training.

The adoption of new methodologies, processes, and technologies tends to be unbalanced depending on the age of the employees. As a rule, with some exceptions, this process tends to be more troublesome for those who have been in the organization for a longer period of time, as it pushes them out of their comfort zone. On the other hand, people who have joined the organization more recently, even if they find it easier to incorporate these methodologies, processes, and technologies, still have to internalize the culture and values that experience brings. To facilitate intergenerational cohabitation, the concept of reverse mentoring (Chaudhuri and Ghosh, 2012), understood as the pairing of a younger, junior employee, who acts as a mentor to share his/her technological skills with an older person with extensive experience in a company, becomes important. This solution can also help to create an adequate organizational climate, where the eldest members share their experience in the organization, while the youngest share their technological skills.

In today’s knowledge society, education plays a decisive role in the transfer of scientific and technological knowledge, as well as analytical and professional skills (Gylfason, 2001). The incorporation of the possibilities brought by ICTs in Higher Education Institutions (HEIs) is leading to the development of new strategic options using policies and plans according to the new demands of the labour market. Therefore, new learning models must be developed where both students and professors must acquire and develop new skills (Bryndin, 2019). Furthermore, HEIs are facing a disruptive scenario, with the emergence of new business models within the training sector. These models need to meet the needs of both external and internal stakeholders, seeking their commitment and improving their experience in the organization. These changes results in the DT of a HEI (Arango Serna et al., 2018).

Almaraz et al. (2017) define the DT of HEIs as the process of technological, cultural and organizational change induced in these institutions by the development of digital technologies. It is not a matter of technology, but of people, values, systems and organizational structures, that must adopt a new model, challenging the previous ideas and assumptions. The DT of the education system is occurring in a disruptive way due to major technological developments and has been critically impacted and boosted by the requirements caused by the COVID 19 pandemic (Livari et al., 2020).

This paper aims at disentangling the intellectual knowledge structure of the research related with the digital transformation and/or digitalization of HEIs. Bibliometric analysis is a useful tool to identify development lines, novel applications, research priorities and references within a topic, according to their geographical location and research network (Wang et al., 2014). This kind of techniques allows academics to analyse a research area considering the citations, co-citations, geographical distribution and word frequency. They provide the researchers with different tools to assess the academic productivity, its impact and its relative influence; to define the intellectual structure of the research topic as well as its evolution; and to identify the different subtopics and its conceptual framework.

We can sum up these goals in some research questions, gathered in Table 1. Questions 1 to 4 have been answered carrying out a productivity analysis. In order to solve questions 5 and 6, following a bibliometric mapping approach, the VOS-Viewer software (Van Eck and Waltman, 2010) has been used to detect the structure of the research topic. The results are presented in the form of visual networks through the analysis of co-citations (documents, authors and journals) and co-words.

**Table 1:** Bibliometric methods used to analyze the research topic.

Techniques	Objective	Research questions	Bibliometric method	Analysis
*Evaluative techniques: SCI-mat*	*(1) To assess academic impact and relative influence*	RQ1. Historical evolution of the literature	*Productivity measures*	Historical evolution of publications
		RQ2. Most productive journals		Distribution of articles by journal
		RQ3. Most productive authors		Distribution of articles by author
		RQ4. Most prominent documents	*Impact metrics*	Citation analysis
*Relational techniques: VOS Viewer*	(2) *To determine intellectual structure*	RQ5. Main documents influencing the intellectual structure	*Co-citation*	Co-citation analysis: documents
	(3) *To identify thematic organization*	RQ6. Main journals around which the research topic of is organized		Co-citation analysis: journals
	(4) *To identify conceptual structure*	RQ7. Patterns and hot topics	*Co-occurrence*	Co-word analysis

The main contribution of this article is to provide a synthesis of the body of research on the DT of HEIs. The relevance of this article lays on the importance of the research topic, which has increased in the current scenario caused by the emergence of the pandemic generated by COVID-19. The lockdown experienced by the population worldwide and the need, within the education sector, to continue with the process of remote learning through digital channels and tools, has led to the massive use of new methodologies that could facilitate remote apprenticeship. This situation has given rise to an opportunity to investigate the impact of DT on the educational process.

Though the previous literature has studied the process of DT in many fields, and even in HEIs, either it has carried out literature reviews (Benavides et al., 2020), or it has focused on aspects like sustainability (González-Zamar et al., 2020) or specific technologies (Reis-Marques et al., 2021). Therefore, there is a need to study and analyse the knowledge structure of this research field.

## Materials and methods

Bibliometric analysis is a scientific field within scientometrics, which applies mathematical and statistical methods to scientific literature with the aim of studying and analysing scientific activity (White and McCain, 1998). Bibliometric methods are frequently used to assess the evolution of a given research area (Liao et al., 2018), analysing a specific scientific domain through bibliographic data through two main approaches: performance analysis and science mapping (Cobo et al., 2011). Amongst the advantages of bibliometric methods, we can state that (i) they present an overview of the scientific literature; (ii) they can generate a more objective summarization of the selected scientific papers than traditional techniques (e.g., literature review); and (iii) they are catching a growing attention of the scientific community (Corsini et al., 2019). Therefore, bibliometric analysis is a powerful tool to study a specific research topic evaluating citations, geographical distribution, co-citations and word frequency.

The objective of this study is to analyse the research trends about the DT process of HEIs. In order to do so, the first step is the selection of the existing academic literature dealing with this topic. The Web of Science (WoS) bibliographic database has been chosen for this purpose, as it is amongst the most relevant ones in the field of Social Sciences, particularly in business and education. Moreover, this database is commonly used in bibliometric analyses in the fields of management and organization.

In order to choose the publications that would be included in the bibliometric analysis and to avoid the subjectivity of the researchers in the data collection, a keyword search of the literature was performed, looking for articles published in peer-reviewed journals. Boolean logical connectors were used in a search string with the terms, [“digital transformation” OR digitalisation OR digitalization] AND [universit* OR “higher education” OR college]. The search for papers was conducted in February 2022 for the period from 1900 to 2021. A later selection eliminated part of the results, keeping only published and early access articles, granting a peer review selection process that ensures their scientific quality. The initial number of documents was 898. After a filtering process performed by the entire research team, which eliminated duplicated articles as well as those ones not directly related with the object of study, a total of 469 articles were finally chosen for the bibliometric analysis (Table 2).

**Table 2:** Data collection.

Features	
Geographic scope	Global scientific production
Database	Web of Science (WoS)
Search criterion	Topic
Kind of document	Article (published or early access)
Time range	1900–2021
Search date	8-feb-22
Keywords	TS = (digital transformation OR digitalization OR digitalization) AND (universit* OR higher education OR college)
Number of initial documents	898
Filter criterion	Duplicated documents. Papers not related with the topic
Number of final documents	469

Initially, Scimat software (Cobo et al., 2012) has been used in order to carry out a productivity analysis, studying the historical evolution of the publications (RQ1), the most relevant journals where they have been published (RQ2), the most productive authors (RQ3) and the most prominent documents in the field (RQ4). Relational techniques, through a bibliometric mapping approach, have been used to determine the intellectual structure of the field of knowledge, regarding the main documents, authors and journals which are the foundations of this literature (RQ5 and RQ6). Finally, the co-word analysis provides an insight into the main themes and research trends, studying the most frequent keywords (RQ7). VOS Viewer software, developed by Van Eck and Waltman, “pays special attention to the graphical representation of bibliometric maps. The functionality of VOS Viewer is especially useful for displaying large bibliometric maps in an easy-to-interpret way” (Van Eck and Waltman, 2010).

## Results

### Evaluative techniques

Evaluative techniques analyse the relative influence and academic impact of a topic. Amongst them, we can find different productivity measures, such as the evaluation of the historical evolution of the number of publications, the distribution of the papers by area, journal and author and the analysis of the most cited papers (Hall, 2011). These measures drop an accurate image about the relative maturity of a research field (Keathley-Herring et al., 2016). These analyses have been carried out using SciMAT software (Cobo et al., 2012), as well as the information generated by WoS itself.

#### Measures of productivity

**Figure 1:**
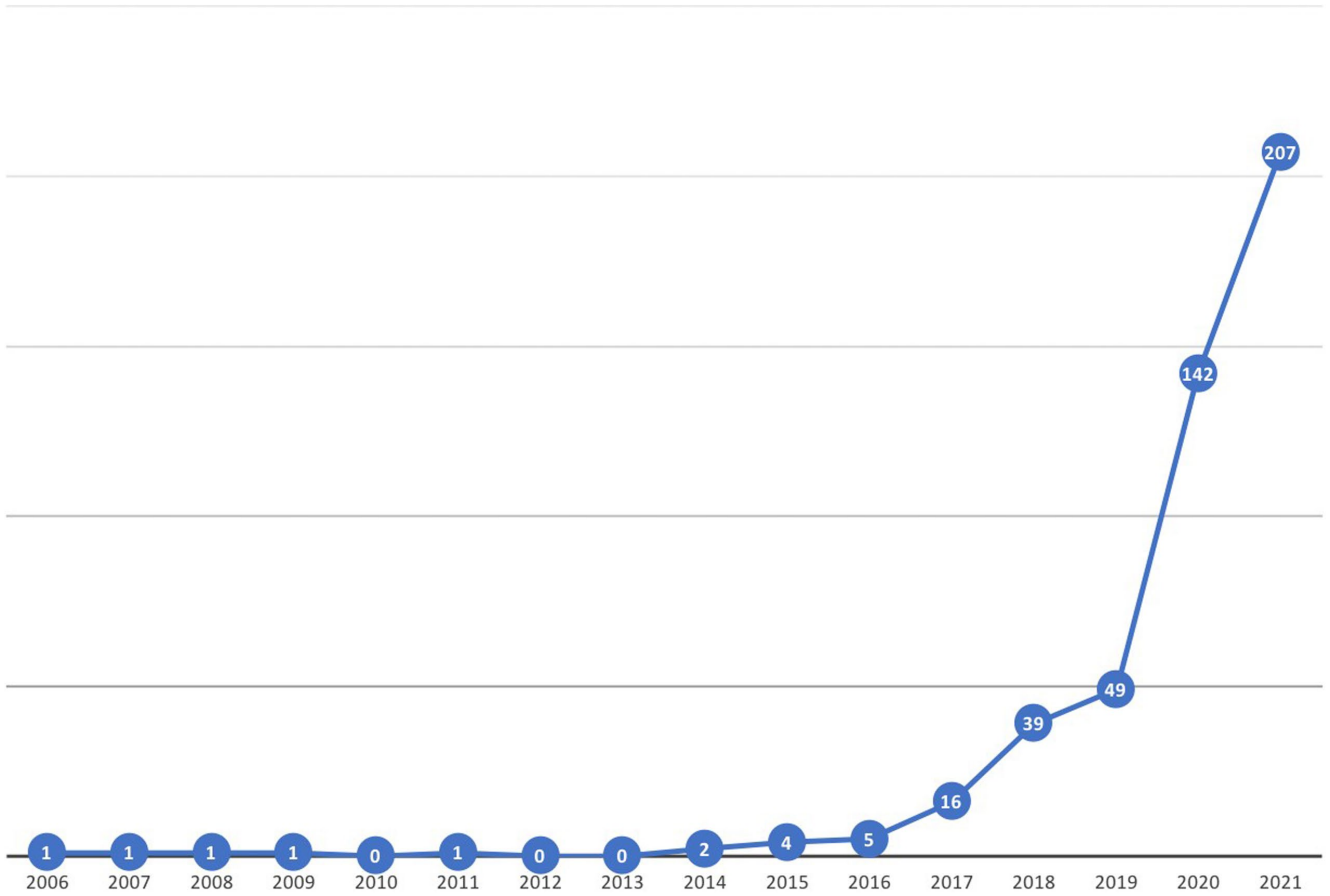
Historical evolution of publications.

Figure 1 shows the total number of papers published in the field of study for the period from 2000 to December 2021. The first publications in this topic appeared in 2006. As it can be seen in the graph, there has been little concern in the literature until 2017, when we can witness a first increase in the number of academic publications. In 2020, a major boost in the literature took place, which has continued in 2021 with 207 articles being published. Between 2020 and 2021, 349 papers about the topic have been published, which means a 74.4% out of the selection of 469.

Analysing this evolution, we can assume that the pandemic generated by COVID-19, with the consequent adoption of remote and blended teaching methodologies, has been a critical factor in the acceleration of the DT of HEIs and, therefore, has generated a dramatical increase in the scientific production about the topic. This evolution also reveals that we are facing a relatively immature research area.

According to WoS, as Table 3 reflects, most of the papers of this selection (197, 42.0%) have been published in the knowledge area of Education and Educational Research. The crisis generated by COVID-19, along with the need to adopt new teaching methodologies which could give the students a core role in their own learning process, has boosted the adoption of ICTs by HEIs and, with this new student-based focus. Some other relevant areas are Business Economics (13.4%), considering the DT of HEIs a way to compete against other institutions; and Science Technology Other Topics, Computer Science and Information Science & Library Science, more focused on the ICTs’ side of DT. So, apparently there are three perspectives to board the DT of HEIs: educational, managerial and technological.

**Table 3:** Distribution of articles by research area.

Research areas	Articles	Percentage (N/469)
Education. Educational Research	197	42.0%
Business Economics	63	13.4%
Science Technology Other Topics	47	10.0%
Environmental Sciences Ecology	31	6.6%
Computer Science	26	5.5%
Information Science Library Science	26	5.5%
Engineering	23	4.9%
Social Sciences Other Topics	18	3.8%
Psychology	12	2.6%

The 469 papers selected were included in 291 publications. Specifically, 218 (74.9%) of them published just 1 article; 38 (13.1%) published 2 articles; 16 (5.5%) published 3 articles and 19 (6.5%) published more than 3 articles. Journals with 6 or more papers on the research topic are listed in Table 4.

**Table 4:** Distribution of articles by journal.

Journal	Articles	Impact factor (2020)/categories
Sustainability	28	0.56 (JCI) Environmental Studies (Q2) Green and Sustainable Science and Technology (Q3)
Education and Information Technologies	15	1.82 (JCI) Education & Educational Research (Q1)
International Journal of Emerging Technologies in Learning	10	1.06 (JCI) Education & Educational Research (Q2)
Education Sciences	8	1.03 (JCI) Education & Educational Research (Q2)
European Journal of Contemporary Education	8	1.18 (JCI) Education & Educational Research (Q2)
Information Technologies and Learning Tools	8	0.36 (JCI) Education & Educational Research (Q3)
International Journal of Computer Science and Network Security	6	0.11 (JCI) Computer Science, Information Systems (Q4)

Apart from Sustainability, a journal mainly focused on Environmental Sciences, which frequently deals also with economic and managerial aspects, the most popular publications are associated with the three main perspectives identified: education (Education Sciences, European Journal of Contemporary Education); management (Business Horizons) and ICTs (International Journal of Computer Science and Network Security). There are also some relevant journals placed in the intersection of these areas, mainly combining educational and technological perspectives, such as Education and Information Technologies, International Journal of Emerging Technologies in Learning or Information Technologies and Learning Tools.

There is a total of 1,472 authors of the 469 papers. The highest proportion of authors (96.2%; *n* = 1,416/1,472) is related to only one publication, while just a 3.8% (*n* = 56/1,472) have taken part in two or more publications. Table 5 presents the most prolific authors.

**Table 5:** Most prolific authors in the topic.

Rank	Name of author	Country of author	University/institution	Number of publications
1	Aditya, B.R.	Indonesia	Telkom University	4
2	Garcia-Penalvo, F.J.	Spain	University of Salamanca	4
3	Abad Segura, E.	Spain	University of Almería	3
4	González Zamar, M.D.	Spain	University of Almería	3
5	Akhmetshin, E.M.	Russia	University of Kazan	3
6	Djakona, A.	Latvia	ISMA University of Applied Sciences	3
7	Ferdiana, R.	Indonesia	Universitas Gadjah Mada	3
8	Kholiavko, N.	Ukraine	Chernihiv Polytechnic National University	3
9	Vasilev, V.L.	Russia	Kazan Federal University	3
10	Zawacki-Richter, O.	Germany	FernUniversität in Hagen	3

The results reveal the presence of different research teams specialized in the analysis of the effect of technologies in education, and sometimes more specifically in HEIs. The work of Aditya (Telkom University, Indonesia), who has sometimes collaborated with Ferdiana (Universitas Gadjah Mada, Indonesia), is especially focused on the implementation of technological teaching methodologies, such as virtual classroom. García-Penalvo (University of Salamanca, Spain) has delivered a long research trajectory dealing with e-learning, which has become a major concern due to the pandemic derived from COVID-19.

The works of Abad-Segura and González-Zamar (University of Almería, Spain) have frequently dealt with the environmental foundations of the digitalization of HEIs, as well as the role played by ICTs on creativity. Kholiavko (Chernihiv Polytechnic National University, Ukraine) has published different papers (occasionally working with Djakona, from ISMA University of Applied Sciences, Latvia) related with the impact of DT in economic development, being HEIs critical in that relationship. Akhmetshin (University of Kazan, Russia) analyses the needs and steps in the creation of a digital university. The work of Vasilev (Kazan Federal University, Russia) deals more frequently with management control systems in a digital context. Finally, Zawacki-Richter (FernUniversität in Hagen, Germany) has published several works analysing specific aspects of the implementation of ICTs in HEIs, using literature reviews and bibliometric analyses.

#### Measures of impact: Citation analysis

The most prominent papers in a research area are those with the highest levels of citation. Thus, citation analysis allows us to detect the influence of certain documents on a particular topic. Table 6 shows the most cited papers amongst the 469 selected ones, ordered considering the number of citations per year.

**Table 6:** The 10 most frequently cited publications in DT of HEIs.

R	Title	Authors	Country (1st aut)	Journal	TC	(C/Y)
1	Digital transformation of everyday life – How COVID-19 pandemic transformed the basic education of the young generation and why information management research should care?	Livari et al. (2020)	Finland	International Journal of Information Management	100	50
2	The future of business education: A commentary in the shadow of the Covid-19 pandemic	Krishnamurthy (2020)	United States	Journal of Business Research	65	32.5
3	Online Assessment in Higher Education in the Time of COVID-19	García-Peñalvo et al. (2020)	Spain	Education in the Knowledge Society	65	32.5
4	Sustainable Management of Digital Transformation in Higher Education: Global Research Trends	Abad-Segura et al. (2020a)	Spain	Sustainability	46	23
5	Digital transformation in German higher education: student and teacher perceptions and usage of digital media	Bond et al. (2018)	Germany	International Journal of Educational Technology in Higher Education	73	18.25
6	The COVID-19: the enzyme of the digital transformation of teaching or the reflection of a methodological and competence crisis in higher education?	García Peñalvo and Corell (2020)	Spain	Campus Virtuales	34	17
7	COVID-19 as an accelerator for digitalization at a German university: Establishing hybrid campuses in times of crisis	Skulmowski and Rey (2020)	Germany	Human Behavior and Emerging Technologies	35	17.5
8	COVID-19 and the Digital Transformation of Education: What Are We Learning on 4IR in South Africa?	Mhlanga and Moloi (2020)	South Africa	Education Sciences	32	16
9	The COVID-19 Pandemic as an Opportunity to Foster the Sustainable Development of Teaching in Higher Education	Sá and Serpa (2020)	Portugal	Sustainability	26	13
10	The glocal curriculum: A model for transnational collaboration in higher education for sustainable development	Caniglia et al. (2018)	Germany	Journal of Cleaner Production	26	6.5

Given that most papers have been published in 2020 and 2021, the influence and presence of COVID-19 in the academic literature about the DT of HEIs could be expected. The most cited article (100 times since its publication), Livari et al. (2020) analyzes the DT of children education driven by the pandemic, as well as the changes that higher education will have to face to adapt to the needs of this new challenging generation. Krishnamurthy (2020) has focused his study on the impact in the university, the business world and the students at business schools derived from COVID-19, that lead to a massive presence of technologies. García-Peñalvo et al. (2020) analyse the guidelines given by The Group of Online Teaching Managers of the Public Universities of a Spanish region in order to adapt the face-to-face evaluation methods to remote ones, due to the curfews and lockdowns derived from the pandemic. In another paper, García Peñalvo and Corell (2020) wonder if COVID-19 has meant a triggering event to accelerate the DT of Spanish HEIs or, in fact, has revealed previous weaknesses in the adoption of European Higher Education System requirements dealing with an education more focused on competences than on contents. Skulmowski and Rey (2020) analyse the quick process of digitalization that had to be faced by German Universities, proposing a strategy of hybrid campuses that could be useful for potential future emergencies. Mhlanga and Moloi (2020), in turn, analyse how COVID-19 could have meant a turning point in South African education system, which traditionally had physical space problems, as it pushed the transition to virtual and remote practices. Finally, Sá and Serpa (2020) also consider the challenges and opportunities brought by the pandemic to walk towards an increase in the sustainable development of teaching practices.

Nevertheless, there are three articles amongst the most cited ones which are not directly related with the pandemic. Abad-Segura et al. (2020b) carry out a bibliometric analysis about the sustainable management of DT of HEIs, stressing one of the main contributions of industry 4.0, a boost for sustainability. In fact, the role of these changes, materialized in the creation of a *glocal* (global but local) curriculum, is presented by Caniglia et al. (2018) as an opportunity to foster sustainable development. Finally, Bond et al. (2018) analyse the choices and preferences of both teachers and students in the DT of German Universities.

### Evaluative techniques

Relational co-citation and co-word analysis techniques reveal the intellectual structure of the research topic, showing the main themes addressed (Zupic and Cater, 2015). A bibliometric mapping tool, the VOS-Viewer software, is used to provide a visual description of the analysis (Van Eck and Waltman, 2010).

#### Co-citation analysis

Co-citation analysis aims at the identification of interrelated influential works and consequently, the intellectual structure and thematic organization of a research topic. Two items are co-cited when they are cited together in another article, which means that there is thematic similarity and affinity between them. Depending on the unit, different types of co-citation analyses have been carried out in this article: document co-citation, journal or source co-citation and author co-citation.

**Figure 2:**
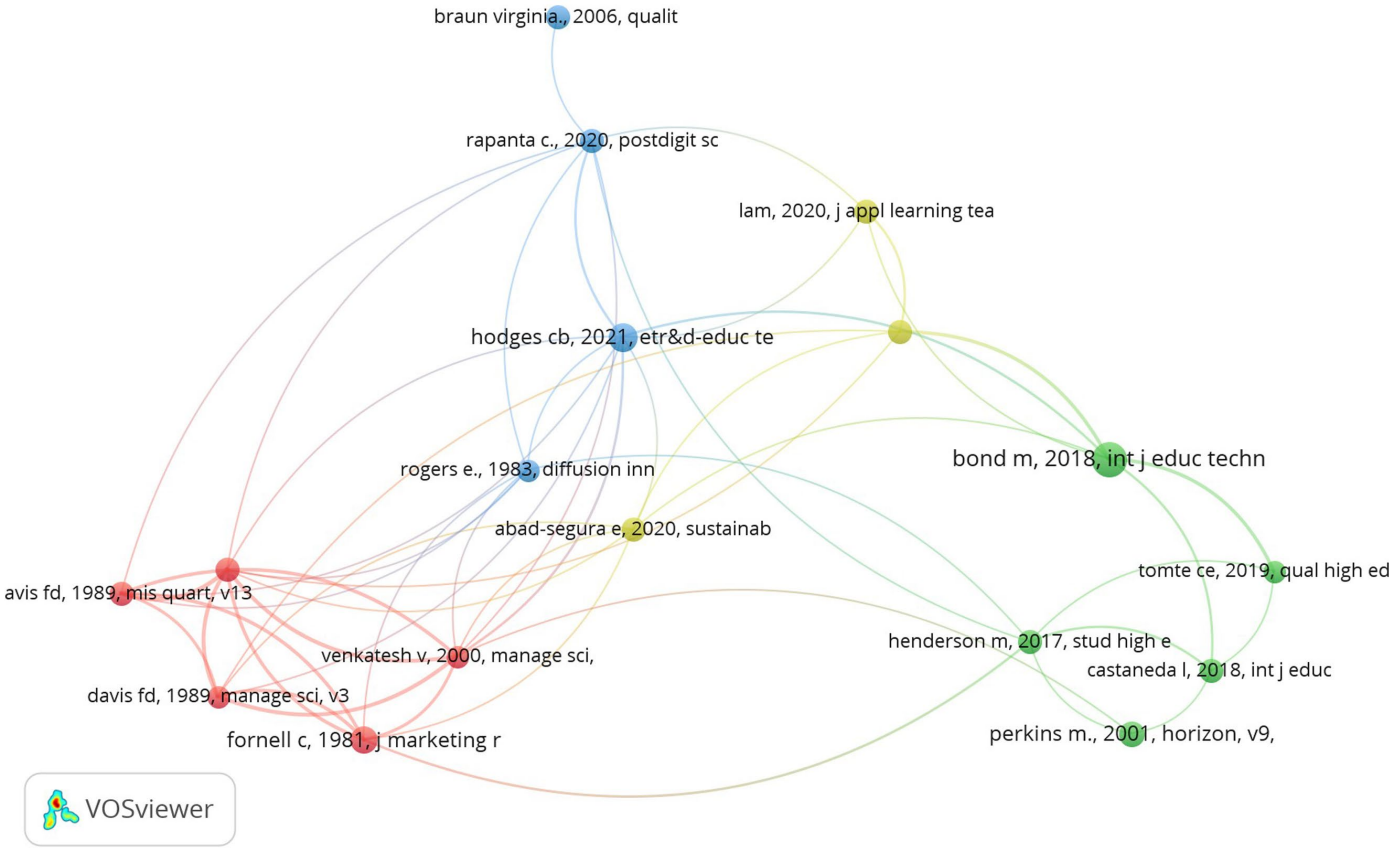
Co-citation of documents in the field of DT in HEIs.

Document co-citation analysis allows us to identify the studies that are most frequently cited together (Small, 1973). We identified 16,288 cited references in the 469 papers selected. 17 of them met the minimum threshold of being cited in at least 7 papers. Figure 2 shows the result of this analysis. The dots or nodes illustrate the references: the greater the number of citations per document, the larger the node size. The links between the nodes represent co-citation relationships. The strength of each link is illustrated by its thickness.

According to the method used by VOS-Viewer, we have found four clusters. The red one includes five references mainly related with the adoption of technologies. Venkatesh and Davis (2000), as well as Venkatesh et al. (2003), aim at enlarging the technology adoption model (TAM), which is a classical foundation when studying the adoption on technological innovations in different contexts. Two other works of Davis et al. (1989) and Davis (1989) deal also with the drivers of technological acceptance. Finally, Fornell and Larcker (1981) is mainly a methodological paper which provides academics with guidelines in order to carry out survey research in operations management.

The green cluster is composed of five papers, being one of them Bond et al. (2018), which deals with the use of specific technologies by some of the most relevant stakeholders involved in this process, professors and students. Henderson et al. (2017) and Tømte et al. (2019) share the same goal of achieving a deeper understanding of the reasons underlying the higher level of adoption of specific technologies amongst students. Castañeda and Selwyn (2018) propose that maybe it is not specific tools, but their integration and use, which provides a HEI with better learning results. Finally, the seminal work of Prensky (2001) analyses the different relation with ICTs of digital natives and digital immigrants.

The yellow cluster includes just three works which could be associated with the management of the implementation of DT of HEIs. While Matt et al. (2015) directly study the DT strategies of universities and colleges, Abad-Segura et al. (2020b) work is specifically focused on the sustainable management of the process. Crawford et al. (2020) board the organizational impact of the changes required by the DT of HEIs.

Finally, the blue cluster is the less homogenous one, as it includes works which are not especially aligned with each other but are fundamental references for the rest of the papers. Hodges (2021) tries to add a practical perspective to the previous work of Hilton (2016), reacting to the crisis generated by COVID-19. Braun and Clarke (2006) deliver a methodological paper which explains the use of thematic analysis in psychology. Rapanta et al. (2020) are also concerned with the impact of COVID-19 on HEIs. Finally, Rogers and Williams (1983) is a classical reference when analysing the diffusion of innovation.

The journal co-citation analysis also contributes to the study of the thematic organization of a research field (McCain, 1990). Two sources are normally cited together when there are similarities in their research areas. In our sample of 469 documents, a total of 9,960 cited sources were identified, out of which 18 met the threshold of a minimum of 45 citations.

**Figure 3:**
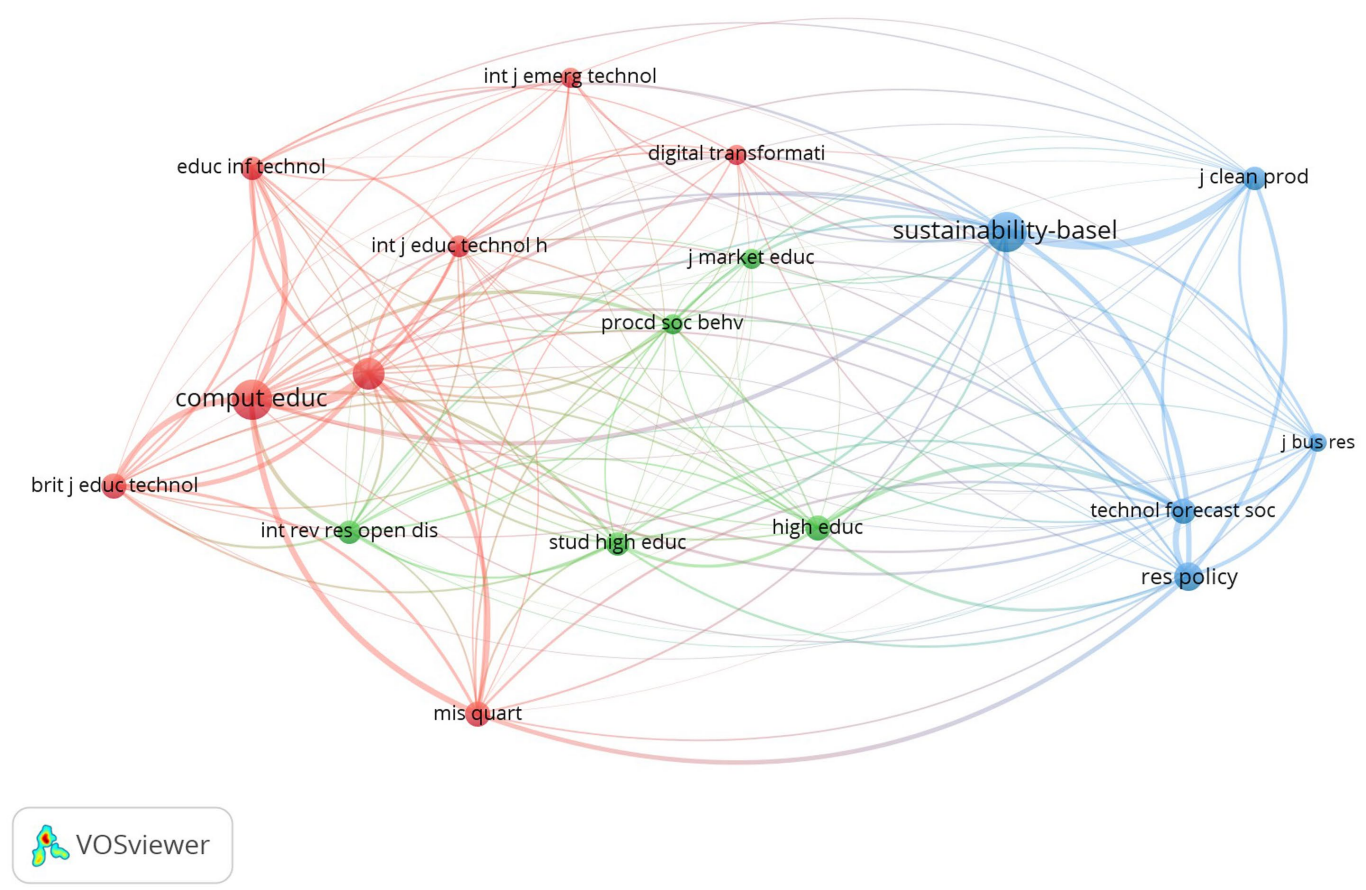
Co-citation network of journals in the field of DT in HEIs.

Figure 3 shows the existence of three main groups of journals referenced by the literature about the DT of HEIs. The red cluster, which joins the highest number of citations, gathers publications that belong directly to this same research area, that is, that deal with the use of technologies in education, such as Computers & Education, Education and Information Technologies or the British Journal of Educational Technology; along with some other ones more directly related with ICTs and systems, being MIS Quarterly a reference in this field. The blue cluster puts together some journals related with the social impact of the DT of HEIs (Sustainability, Journal of Cleaner Production, Technological Forecasting and Social Change) with some others more closely linked with research as a critical activity of HEIs (Journal of Business Research, Research Policy). Placed between them, we can find the green cluster, which includes publications linked with the management of HEIs (Studies in Higher Education, Higher Education and even the Journal of Marketing Education).

#### Co-word analysis

**Figure 4:**
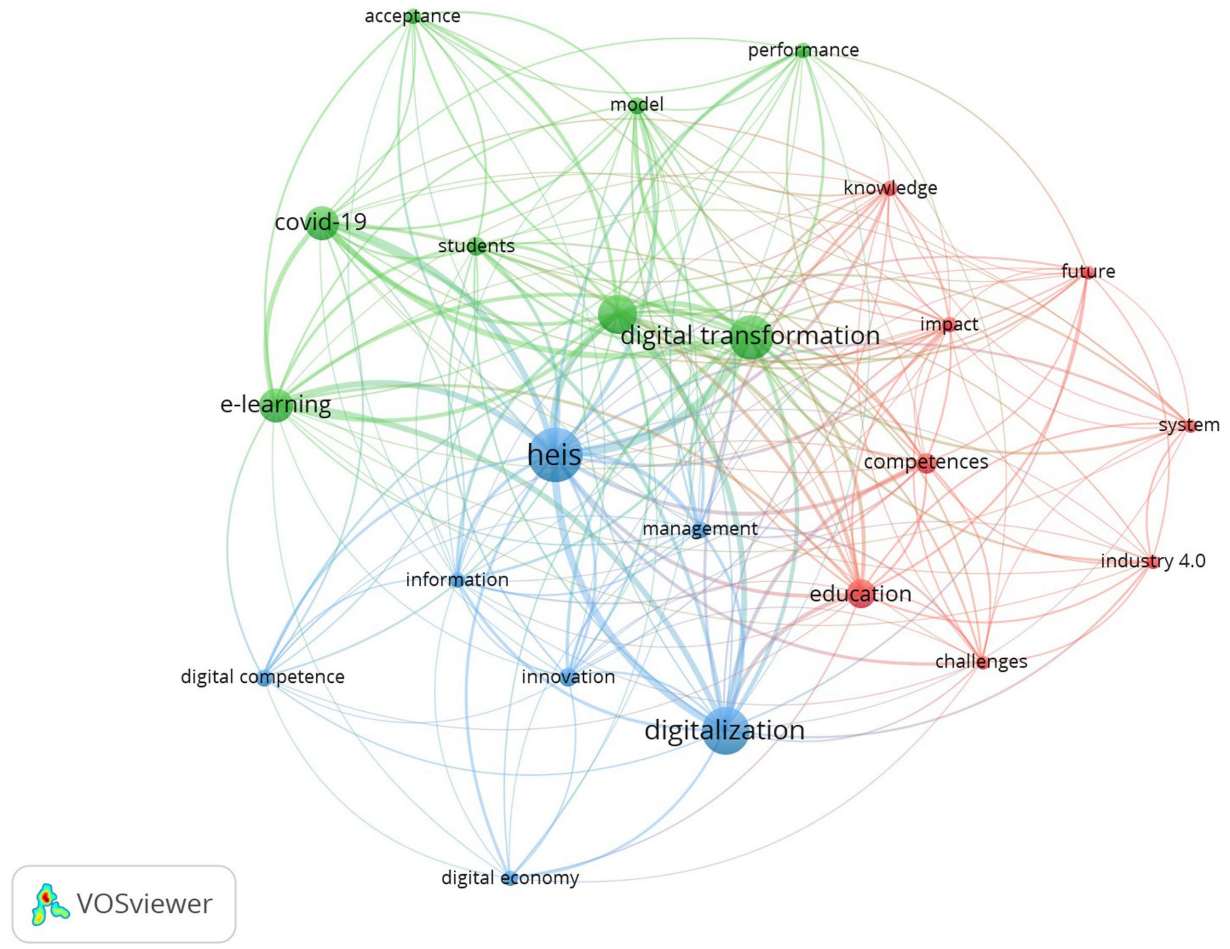
Co-occurrence network of keywords in the field of DT in HEIs.

Research trends and hot topics emerge from the co-word analysis of the most frequent keywords (Li et al., 2016). As can be seen in Figure 4, in our sample of 469 papers, we detected 1,774 keywords. We only considered the 23 keywords that appear in at least 12 publications. The nodes illustrate the occurrence of the keywords, while the links between the nodes represent the number of times that the words appear together.

The main keywords in the research topic are: “HEIs” (167 occurrences and 332 total link strength), “digital transformation” (112 occurrences and 234 total link strength), “digitalization” (132 occurrences and 203 total link strength), “ICTs” (86 occurrences and 202 total link strength) and “COVID-19” (69 occurrences and 139 total link strength). Apart from the somehow obvious ones, we can find again reflected the earthquake generated by COVID-19 in the entire sector.

The map reflects three different moments dealing with the topic. Technologies (blue cluster) appear as the initial and basic component of a first step, the “digitalization” of “HEIs,” when they tiptoe into the “digital economy” using “innovations” in the “management” of the “information” of the organization, which requires the acquisition of “digital competences” by their members. The green cluster shows how “COVID-19” has forced a massive “acceptance” of “ICTs,” accelerating the process of “digital transformation” of universities, which had to provide their “students” with adequate solutions to facilitate their “e-learning” experience. Finally, the red cluster deals mainly with the “future” and the “challenges” for the “education” sector as a part of “industry 4.0,” which will require the creation of “systems” to manage and spread the main product of HEIs, “knowledge” creation and “competences” development.

## Conclusion

Most of baby boomers and X-gen members are witnessing and actively taking part in a truly revolutionary change in the way we experience nearly every aspect of our lives. The appearance of different radically innovative ICTs (personal computers, the Internet, social media, smartphones…) have changed our interactions with each other, how we make businesses, our leisure activities and the very nature of our jobs.

Education plays a critical role in these changes. On the one hand, all these technologies have also entered schools, colleges and universities, changing the way we learn and teach nearly any subject. On the other hand, these institutions will provide the new generations with the adequate knowledge, tools and capabilities to profit from these ICTs, squeezing their future possibilities of additional disruptive changes.

Digitalizing means just a first step in the process, in which the institutions use new tools in order to carry out old activities. Further than just using technologies, digitally transforming an educational institution, or even the entire system, requires a redesign of the learning and teaching practices, reconsidering the roles of all the stakeholders involved in the educational process, promoting practices like flipped learning, gamifications or crossover learning; as well as the implementation of new managerial practices where ICTs reshape the main value activities of these organizations.

The process of DT of academic institutions experienced a complete shock when COVID-19 came into the scene. All the agents were required a maximum level of creativity in order to face this major challenge. Some practices that had been just pilot programs before 2020 became suddenly the only possible way to keep on working.

The literature about the digitalization and DT of HEIs has reflected this shocking event. Though there had been a previous growth in the academic production after 2017, its evolution would have more than likely been slower if COVID-19 crisis had not turned everything upside down. The number of publications in 2020, 142, is clearly higher than the total previously cumulated scientific production. 2021 has not meant a change in this trend, and the number of new papers has broken the barrier of 200. This exponential growth allows us to set a first conclusion: probably spurred by COVID-19, the DT of HEIs is unstoppable, and it is obviously here to stay. The scientific community has not ignored this reality, increasing the attention paid to this phenomenon.

The interest about the digitalization and DT of HEIs is not just a technological issue, but also a managerial, and especially educational, concern. Therefore, despite the importance of technical research areas (Computer Science, Engineering), nearly half of the papers analysed were published in journals linked with Educational Research, while Business Economics publications are also importantly represented. Finally, the positive impact of remote learning in sustainable development has also been reflected by the academic literature.

The theoretical foundations of the studies dealing with the DT of HEIs are related with these areas, technologies, education and management. Nevertheless, some other groundings, even philosophical ones, must be considered, as we might well be witnessing the birth of a new era which challenges many aspects related with how we conceive everything. As it has been stated before, education is necessarily a critical lever of such changes. The co-word analysis has clearly reflected the existence of a past (productive use of ICTs in HEIs), a present (the adaptation of the teaching and learning practices to the needs of the stakeholders after the burst of COVID-19) and a future (building new complete educational systems adapted to the needs of the information society) of the DT of colleges and universities.

This paper makes a relevant contribution to the state of the art, disentangling the knowledge structure of the research in this field, as well as stressing the increasing importance of this research area. The DT of HEIs is currently taking part in any organization, affecting and being affected by the needs, goals and positions of many relevant stakeholders, such as the students, the professors, the administrative staff, governments and authorities, economic agents and, last but not least, the entire society. The DT of HEIs is directly related with the achievement of one of the SDGs, #4, a Quality Education, but is undeniably a cornerstone when trying to reach any other one.

Any bibliometric analysis has to face some limitations due to its very nature, being the selection of documents to be analysed the main one. Web of Science includes the vast majority of the most prominent research in this field, though some relevant and promising studies could have been published in journals which are not indexed by WoS. The choice of the search string of the authors of this paper, combined with the selection of the keywords in the articles analysed, could have excluded specific documents from the selection. Some of the papers considered may not include keywords, influencing the results of the co-word analysis. Finally, the subjectivity of the authors of this paper cannot be completely avoided when analysing the results.

Though some of the previous research has been directly related with the implementation and adoption of some specific technologies, this is, in our opinion, a limited path. As long as the DT is here to stay, probably the most promising aspects that are being studied, and will generate more scientific dialog in the future, is the perception of the DT transformation of HEIs by the different stakeholders involved in this change (managers, professors, administrative staff, students, employers, society as a whole…), as they hold very different points of view, but probably all of them are necessary to squeeze all the potential of this technological revolution.

COVID-19 crisis has been a tragedy for mankind. It has destroyed lives and caused severe damages to many people. It has also questioned the way we were carrying out many of our tasks, sometimes considering there was no other option. Probably one of the most affected aspects has been education. But crises normally also bring the chance to rebuild and reshape. The digital transformation of universities and colleges brings our society the opportunity to do something good out of the devastation. All the agents involved have recognized this turning point and are doing their best to deliver a new and better education for the generations to come, based on the potential of ICTs, in a process that has just started. The education of the future will be more technological, connected and adapted to the needs of a brand new society.

## Data availability statement

Publicly available datasets were analyzed in this study. This data can be found at: Web of Science.

## Author contributions

VD-G and RG-L: conceptualization and resources. RG-L and AM-N: writing—original draft preparation and supervision. J-LR-S and VD-G: writing—review and editing and visualization. J-LR-S and AM-N: methodology and formal analysis. J-LR-S, VD-G, AM-N, and RG-L: validation. All authors contributed to the article and approved the submitted version.

## Conflict of interest

The authors declare that the research was conducted in the absence of any commercial or financial relationships that could be construed as a potential conflict of interest.

## Publisher’s note

All claims expressed in this article are solely those of the authors and do not necessarily represent those of their affiliated organizations, or those of the publisher, the editors and the reviewers. Any product that may be evaluated in this article, or claim that may be made by its manufacturer, is not guaranteed or endorsed by the publisher.
