# Corrigendum: Digitalization and digital transformation in higher education: A bibliometric analysis

**DOI:** 10.3389/fpsyg.2023.1154578

**Published:** 2023-02-22

**Authors:** Vicente Díaz-García, Antonio Montero-Navarro, José-Luis Rodríguez-Sánchez, Rocío Gallego-Losada

**Affiliations:** ^1^Facultad de Ciencias Jurídicas y Sociales, Universidad Rey Juan Carlos, Paseo de los Artilleros, Madrid, Spain; ^2^ESIC Business & Marketing School, Pozuelo de Alarcón, Madrid, Spain; ^3^Department of Business Economics (Adm., Dir. and Org.), Applied Economics II and Fundamentals of Economic Analysis, Facultad de Ciencias Jurídicas y Sociales, Universidad Rey Juan Carlos, Paseo de los Artilleros, Madrid, Spain; ^4^Department of Financial Economy and Accountancy, Facultad de Ciencias Jurídicas y Sociales, Universidad Rey Juan Carlos, Paseo de los Artilleros, Madrid, Spain

**Keywords:** digitalization, digital transformation, cultural change, bibliometric analysis, resistance to change, research trends, higher education institutions

In the published article, there was an error regarding the affiliation(s) for Vicente Díaz-García. As well as having affiliation(s) 1, they should also have “^2^ESIC Business & Marketing School, Pozuelo de Alarcón, Madrid, Spain.”

The authors apologize for this error and state that this does not change the scientific conclusions of the article in any way. The original article has been updated.

